# Targeting ubiquitin signaling for cancer immunotherapy

**DOI:** 10.1038/s41392-020-00421-2

**Published:** 2021-01-13

**Authors:** Xiaofei Zhou, Shao-Cong Sun

**Affiliations:** 1 Department of Immunology, The University of Texas MD Anderson Cancer Center, 7455 Fannin Street, Box 902, Houston, TX 77030 USA; 2 The University of Texas Graduate School of Biomedical Sciences, Houston, TX 77030 USA

**Keywords:** Tumour immunology, Immunotherapy

## Abstract

Cancer immunotherapy has become an attractive approach of cancer treatment with tremendous success in treating various advanced malignancies. The development and clinical application of immune checkpoint inhibitors represent one of the most extraordinary accomplishments in cancer immunotherapy. In addition, considerable progress is being made in understanding the mechanism of antitumor immunity and characterizing novel targets for developing additional therapeutic approaches. One active area of investigation is protein ubiquitination, a post-translational mechanism of protein modification that regulates the function of diverse immune cells in antitumor immunity. Accumulating studies suggest that E3 ubiquitin ligases and deubiquitinases form a family of potential targets to be exploited for enhancing antitumor immunity in cancer immunotherapy.

## Introduction

Targeted therapy and immunotherapy are two advanced strategies of cancer treatment.^1^ Strategies in targeted therapy are based on cell-autonomous mechanisms of tumor growth and survival to directly target tumor cells. While these methods generate clinical responses in most cancer patients carrying the specific genetic mutations, they often lack durability due to acquisition of resistance by tumor cells.^1^ Immunotherapy does not directly target tumor cells but rather acts through mobilization of patients’ own immune system to attack the tumors. A major advantage of immunotherapy is the possible generation of long-lasting antitumor effects in addition to relatively minor side effects.^2^ In particular, therapies based on immune checkpoint inhibitors (ICIs), monoclonal antibodies targeting the T cell coinhibitory receptors cytotoxic T-lymphocyte-associated protein 4 (CTLA4) and programmed cell death protein 1 (PD1), have revolutionized cancer treatments.^2^ However, since the response rate of many types of cancers to single ICI therapies is still low, extensive efforts are being made to develop combination strategies and characterize new therapeutic targets, such as the costimulatory receptors of T cells. In addition, targeting intracellular signaling molecules provides an additional opportunity for improving antitumor immunity.

Ubiquitination has become a well-recognized signaling mechanism that regulates diverse aspects of immune system functions. Ubiquitination is a posttranslational mechanism of protein modification involved in diverse biological processes, including proteasomal protein degradation, receptor endocytosis, DNA repair, gene transcription, kinase activation, protein-protein interaction and assembly of signaling complexes.^3^ Ubiquitination involves covalent conjugation of monoubiquitin or polyubiquitin chains onto lysine (K) resides of target proteins, which is catalyzed by sequential actions of ubiquitin-activating (E1), ubiquitin-conjugating (E2) and ubiquitin-ligating (E3) enzymes.^4^ Polyubiquitin chain formation involves connection of ubiquitin molecules via different internal K residues or the N-terminal methionine (M1) residue, leading to generation of a large variety of ubiquitin chains with distinct functions.^5^ For example, K48-linked ubiquitin chains target proteins to the proteasome for degradation, whereas K63-linked and M1-linked (also called linear) ubiquitin chains are best known for mediating protein-protein interaction and enzymatic activation in signal transduction. The process of protein ubiquitination is counteracted by deubiquitinases (DUBs), a large family of proteases that cleaves ubiquitin chains.^3^ Mammalian cells express more than 600 E3 ligases and about 100 DUBs, which display substrate specificities and regulate specific cellular functions.^3,5^ An increasing number of E3s and DUBs have been identified as important regulators of immune responses, providing exciting opportunities for developing novel drugs for cancer immunotherapy. In this review, we will discuss recent studies regarding the roles of ubiquitination in regulating immune cell functions and their potential in cancer immunity.

## Ubiquitin regulation of dendritic cell functions

Dendritic cells (DCs) are professional antigen-presenting cells (APCs) that mediate T cell activation and play a vital role in immune responses against infections and cancer.^6^ In tumor microenvironment, DCs uptake antigens released from dying tumor cells and migrate to draining lymph nodes, where DCs present the tumor antigens to the TCR of tumor-specific T cells, triggering T cell activation and differentiation, and the generated effector T cells traffic to the tumors to mediate tumor cell destruction.^7^ Among the different DC subsets, conventional DCs (cDCs) are specialized in antigen presentation for T cell activation, and the cDCs can be further divided into type 1 cDC (cDC1) and type 2 cDC (cDC2) subsets characterized by expression of CD8α and CD11b, respectively.^8^ The cDC1 subset is particularly efficient in presenting cancer antigens to CD8 T cells for mediating anticancer immunity. Understanding the mechanisms that regulate DC functions is crucial for rational design of cancer vaccines and other therapeutic agents to improve the efficacy of cancer immunotherapy.^6,9^

Antigen processing and presentation form an integral part of DC functions, which involves processing of endogenous proteins and internalized exogenous proteins into small peptides and presentation of the peptide antigens by major histocompatibility complex (MHC) molecules.^10^ MHC class I (MHC I) typically presents peptides generated through proteasomal degradation of endogenously synthesized cytosolic proteins. However, MHC I in professional APCs, particularly DCs, can also present peptides from internalized exogenous proteins via a process known as cross-presentation, which is crucial for CD8 T cell responses against infections and tumorigenesis.^11,12^ In this process, the exogenous proteins are either transported from the endosomal vesicles into the cytosol for proteasomal processing or directly processed within the endosomal compartments. MHC class II (MHC II) present peptides derived from lysosomal degradation of extracellular proteins.^10^ The antigens presented by MHC I and II are recognized by the TCR of CD8 and CD4 T cells, respectively. In response to infections or tumorigenesis, DCs undergo a process of maturation, involving upregulation of costimulatory molecules and chemokine receptors, which is required for their migration to the draining lymphoid organs and function in T cell priming.^10^ Ubiquitination plays a crucial role in regulating both antigen processing and maturation of DCs, highlighting new opportunities for DC manipulations in cancer immunotherapy.

## Antigen processing and presentation

The involvement of ubiquitination in MHC I-restricted antigen processing was initially suggested by the finding that fusion of viral proteins with ubiquitin enhances antigen presentation to CD8 T cells.^13^ A more direct evidence came from a study using cells with a temperature-sensitive defect in ubiquitin conjugation.^14^ Similarly, overexpression of a dominant-negative ubiquitin mutant lacking all of the lysine residues in mammalian cells abrogates polyubiquitination and potently inhibits MHC I-restricted processing of endoplasmic reticulum (ER)-targeted proteins.^15^ Accumulating evidence suggests that MHC I-restricted processing of exogenous proteins for cross-presentation involves ER-associated degradation (ERAD),^16–19^ a mechanism mediating retrotranslocation of misfolded proteins from the ER back to the cytoplasm for proteasomal degradation.^20^ ERAD also mediates processing of extracellular proteins involved in antigen cross-presentation.^19^ Thus, approaches to enhance ER entry of protein antigens, such as Grp170 chaperone-based vaccine adjuvant system, improve the efficiency of cross-presentation and induction of CD8 T cell responses in cancer immunotherapy.^19,21^

Ubiquitination has a vital role in ERAD-dependent protein processing. It is thought that ubiquitination of ERAD substrates facilitates their binding by the ERAD molecular machinery required for their retrotranslocation to the cytoplasm.^22^ A major E3 ubiquitin ligase participating in the process of ERAD is the ER-resident transmembrane protein HRD1, which forms a complex with several partner proteins, including SEL1L, HERP, DERLIN-1, OS-9 or XTP3, and BiP, to mediate ubiquitination of ERAD substrates.^15,22^ The E3 action of HRD1 in ERAD is counteracted by a DUB, USP25, which deconjugates the ubiquitin chains from specific ERAD substrates to prevent their retrotranslocation and proteasomal degradation.^23,24^ Another mechanism of HRD1 regulation is through controlling its steady-state levels by ubiquitin-dependent proteasomal degradation, a process that is protected by a DUB USP19.^25^ While HRD1 has been implicated in the regulation of ERAD and MHC I-restricted antigen processing, a recent gene-targeting study suggests that HRD1 also plays an important role in promoting MHC II-restricted antigen presentation to CD4 T cells.^26^ In this function, HRD1 promotes transcriptional expression of the MHC II gene via a mechanism that involves ubiquitin-dependent degradation of B lymphocyte-induced maturation protein 1 (BLIMP1), a transcriptional suppressor known to inhibit expression of the MHC class II transactivator (CIITA).^26^ These studies suggest that manipulating HRD1 expression and ERAD events may be an approach to improve antigen presentation and T cell responses in cancer immunotherapy.

Another E3 ubiquitin ligase regulating ERAD is the ER-resident ring finger protein RNF5.^27^ RNF5, together with the E2 Ubc13, mediates ubiquitination of JNK-associated membrane protein (JAMP), an ER membrane protein facilitating ERAD-dependent degradation of unfolded proteins.^27,28^ RNF5-mediated JAMP ubiquitination inhibits the association of JAMP with ERAD molecular components, thereby limiting ERAD-mediated protein degradation.^27^ Thus, unlike the positive role of HRD1, RNF5 negatively regulates ERAD, although it remains to be examined whether RNF5-mediated ERAD regulation has a role in regulating antigen processing and T cell activation. Nevertheless, a role of RNF5 in regulating antitumor immunity has been linked to a function of RNF5 in regulating another ER-protective process, unfolded protein response (UPR).^29^ UPR is a mechanism that restores normal function of the ER in response to ER stress and has been linked to important functions of immunity and inflammation.^30^ RNF5 deficiency in mice attenuates UPR and reduces antimicrobial peptide expression in intestinal epithelial cells, which in turn alters gut microbiota composition and, thereby, promotes the recruitment and activation of DCs for T cell activation.^29^ Consequently, the RNF5-deficient mice display stronger antitumor immunity in a microbiota-dependent manner. Another immunoregulatory function of RNF5 is to mediate ubiquitin-dependent degradation of stimulator of interferon genes (STING) and negatively regulate antiviral innate immune responses.^31^ Of note, STING is a central DNA sensing component that mediates DC activation in tumor microenvironment and has been actively exploited in cancer immunotherapy.^32,33^ Whether RNF5 deletion in DCs promotes DC activation and T cell priming in antitumor immunity is yet to be investigated.

Ubiquitination also regulates the surface expression and recycling of MHC molecules. Early studies suggest that proteins encoded by some viruses, such as HIV, KSHV, downregulate surface MHC I by inducing its endocytosis and degradation.^34,35^ Ubiquitin provides a signal for MHC I incorporation into intralumenal vesicles of multivesicular bodies (MVBs), a subpopulation of late endosomes fusing with lysosomes.^36^ Members of the membrane-associated RING-CH (MARCH) E3 ubiquitin ligases have been shown to mediate endosomal trafficking and degradation of immune receptors, including MHC molecules.^37^ MARCH4 and MARCH9 mediate MHC I ubiquitination to promote its endocytosis and lysosomal degradation, whereas MARCH1 and MARCH8 mediate ubiquitin-dependent degradation of MHC II.^38,39^ Studies using MARCH1-deficient DCs reveal that although MARCH1-mediated MHC II ubiquitination does not affect MHC II endocytosis, it prevents recycling of the internalized MHC II/peptide complexes and promotes their degradation.^40,41^ Thus, MARCH1-deficient DCs have drastically increased levels of MHC II/peptide complexes on the cell surface. Interestingly, lipopolysaccharide (LPS)-induced DC maturation is associated with downregulation of MARCH1 expression and attenuation of MHC II ubiquitination, coupled with increased MHC II surface stabilization.^41,42^ Conversely, the immunosuppressive cytokine IL-10 induces the expression of MARCH1 and, thereby, downregulates MHC II surface expression, contributing to IL-10-mediated suppression of antigen presentation.^43–45^ The role of ubiquitination in MHC regulation has also been demonstrated through the study of DUBs. Ubiquitin C-terminal hydrolase-L1 (UCH-L1) has been shown to positively regulate MHC I recycling and antigen cross-presentation to CD8 T cells, although it is unclear whether UCH-L1 directly deubiquitinates MHC I.^46^ Another DUB, USP14, facilitates MHC I-restricted direct antigen presentation, particularly the peptides derived from defective ribosomal products (DRiPs).^47^ These findings suggest that targeting E3 ubiquitin ligases and DUBs represents a potential strategy to modulate MHC expression and antigen presentation in vaccine development and other approaches of cancer immunotherapy.

## DC maturation and activation

DCs normally exist in an immature state in peripheral tissues, and their T cell-priming function requires a process of maturation characterized by upregulation of MHC II, costimulatory molecules such as CD80 (also called B7-1) and CD86 (also called B7-2), and chemokine receptors.^48^ DC maturation is stimulated by signals from different immune receptors, including pattern-recognition receptors (PRRs) that respond to pathogen-associated molecular patterns (PAMPs) or host-derived damage-associated molecular patterns (DAMPs).^5,48^ Exposure of DCs to PRR ligands also activate DCs to secrete cytokines that regulate the differentiation of activated T cells to generate specific subsets of effector T cells. DCs and other innate immune cells express several families of PRRs, which include toll-like receptors (TLRs), cytosolic DNA sensors, RIG-I-like receptors (RLRs), NOD-like receptors (NLRs), and C-type lectin receptors (CLRs).^49^ PRR signals function by triggering cascades of signaling events, including activation of MAP kinase (MAPKs), IkB kinase (IKK), and the IKK-related kinase TBK1, which mediate activation of transcription factors AP1, NF-κB, and IRF3, respectively.^5^ The MAPK and IKK pathways are crucial for PRR-stimulated DC activation and proinflammatory cytokine production involved in T cell activation and differentiation, whereas the TBK1 pathway mediates induction of type I interferons (IFNs). In addition to regulating antiviral innate immunity, type I IFNs have a pivotal role in inducing DC maturation and anticancer immunity.^50,51^ DCs lacking type I interferon receptor 1 (IFNAR1) are defective in polyI:C-stimulated maturation in vivo, as shown by impaired induction of costimulatory molecules and MHC II.^50^ On the other hand, type I IFN production in DCs is not required for DC maturation, since nonhematopoietic cells are the main source of type I IFN stimulated by polyI:C.^50^ In fact, DC-conditional TBK1 deficiency promotes DC maturation and anticancer T cell responses due to a negative role of TBK1 in regulating IFNAR1 signaling.^52^ TBK1 acts by phosphorylating and promoting the function of STAT3, a negative regulator of IFNAR1 signaling. Thus, the PRR-stimulated signaling pathways play important, although distinct, roles in the regulation of DC maturation and activation involved in antitumor immunity.

**Fig. 1:**
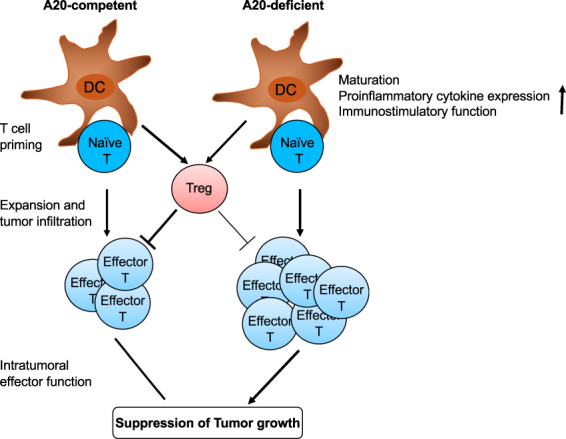
A20 knockdown or knockout in DCs promotes T cell activation and antitumor immunity. DCs with A20 knockdown or knockout display enhanced maturation, proinflammatory cytokine expression and T cell-stimulatory function. A20-silenced DCs are much more potent than wildtype DCs in promoting antitumor T cell responses and suppressing tumor growth

Ubiquitination regulates both the upstream common signaling steps and the downstream specific pathways of the PRR signaling.^5^ Several E3 ubiquitin ligases, including cIAP1, cIAP2, Peli1, TRAF6, TRIM31, and TRIM56, have been shown to conjugate K63-linked polyubiquitin chains to various PRR signaling adapters, such as RIP1, RIP2, TRAF6, MAVS, and STING, thereby facilitating the recruitment and activation of downstream kinases.^5,53–55^ K63 ubiquitination also has a more direct role in regulating downstream signaling events, particularly IKK activation. An early study suggests that ubiquitination of the regulatory subunit of IKK, NF-κB essential modulator (NEMO), is required for DC maturation and activation.^56^ DCs derived from patients with ectodermal dysplasia with immune deficiency (EDI), which carries a point mutation in the C-terminal zinc finger domain of NEMO (C417R), display a defect in CD40-stimulated ubiquitination of NEMO and activation of an NF-κB member, c-Rel, associated with attenuated induction of costimulatory molecules and a key immunostimulatory cytokine, IL-12, as well as T cell-priming function.^56^ A20, an ovarian tumor (OTU) family DUB known to regulate IKK/NF-κB signaling and TNF-induced cell death,^57,58^ has been shown to negatively regulate the maturation, proinflammatory cytokine production, and immunostimulatory functions of DCs^59,60^ (Fig. 1). A20 knockdown in murine and human DCs enhances the induction of costimulatory molecules and proinflammatory cytokines by ligands of TLR3 and TLR4, rendering DCs hyper-active in priming T cell responses (Fig. 1). In line with these studies, gene targeting studies demonstrate that A20 functions as a checkpoint in DC activation and survival, which involves inhibition of LPS-stimulated IKK and MAPK signaling pathways.^61,62^ The A20-deficient DCs undergo spontaneous maturation under homeostatic conditions, characterized by aberrant expression of the costimulatory molecules. Consistently, the peripheral lymphoid organs of DC-conditional A20 knockout mice have an increased frequency of effector/memory-like T cells indicative of spontaneous T cell activation under steady conditions.^61,62^ Signal transduction from TLRs, with the exception of TLR3, relies on a common signaling adapter, MyD88, whereas TLR3 signals through another adapter protein, TRIF.^5^ Deletion of MyD88 in DC-conditional A20 knockout mice does not prevent spontaneous DC maturation or aberrant T cell activation, suggesting that A20 restricts MyD88-independent signals to prevent spontaneous DC maturation and aberrant T cell activation under homeostatic conditions.^62^ However, MyD88 deletion prevents A20-deficient DCs from hyper-production of cytokines, including IL-6 and TNF, that drive T cell proliferation. Therefore, A20 restricts MyD88-dependent signals in DCs to suppress IL-6 and TNF production, thereby preventing aberrant T cell expansion.^62^ A20 knockdown studies reveal that A20 downregulation in DCs impairs their function in stimulating regulatory T (Treg) cells, thereby further enhancing effector T cell responses^59^ (Fig. 1). The potential therapeutic value of targeting A20 has been demonstrated using a mouse model of DC-based cancer therapy.^59^ When adoptively transferred into tumor-bearing mouse, the A20-silenced DCs are much more potent than wildtype DCs in suppressing growth of the established tumors, associated with increased infiltration of effector T cells with enhanced resistance to Treg-mediated suppression^59^ (Fig. 1). These studies suggest that A20 controls DC functions in T cell priming and effector function as well as in Treg regulation, implicating A20 targeting as a potential approach for improving the efficacy of cancer immunotherapy, particularly DC-based therapy (Fig. 1).

The function of NF-κB signaling in DCs is also subject to ubiquitin-dependent regulation at the epigenetic level, as demonstrated through the study of a A20-related DUB, Trabid (also called Zranb1).^63^ Trabid is required for TLR-stimulated expression of two proinflammatory cytokines, IL-12 and IL-23, which in turn are crucial for generation of Th1 and Th17 subsets of CD4 effector T cells. Although Trabid is dispensable for the induction of NF-κB nuclear translocation, Trabid is required for recruitment of NF-κB members, particularly c-Rel and p50, to the promoters of *Il12a*, *Il12b*, and *Il23a* genes. Ubiquitination targets the degradation of Jmjd2d, a histone methylase mediating histone modifications at the Il12/Il23 promoters required for binding by p50 and c-Rel, and the ubiquitin-dependent Jmjd2d degradation is counteracted by Trabid-mediated deubiquitination.^63^ Trabid-mediated DC regulation plays an important role in autoimmune responses. Since IL-12 family of cytokines have an important role in regulating antitumor T cell and natural killer (NK) cell responses,^64^ it is important to examine whether Trabid also plays a role in regulating antitumor immunity.

## Ubiquitin regulation of T cell responses

T cells form a central component of adaptive immune responses against infections and cancer. T cell activation occurs in lymphoid tissues that drain the sites of infections or tumorigenesis, where naïve CD4 and CD8 T cells encounter APCs displaying antigens on MHC II and MHC I, respectively. Upon engagement by the antigen/MHC complex, TCR delivers a primary signal for T cell activation. T cell activation also requires costimulatory signals, which in naïve T cells is primarily mediated through ligation of the costimulatory receptor CD28 by its ligands, CD80 or CD86, on APCs.^65^ T cells also express costimulatory receptors of the TNF receptor (TNFR) superfamily, many of which are induced along with T cell activation and are required for long-lasting T cell responses and generation of effector and memory T cells.^66^ In addition, signals stimulated by cytokines and growth factors contribute to the activation and differentiation of T cells. The TCR and costimulatory signals are opposed by signals from a variety of inhibitory receptors, most notably members of PD1 family of co-inhibitory receptors.^67^ Ubiquitination regulates different aspects of signaling events involved in the activation and function of T cells.

## TCR signaling

**Fig. 2:**
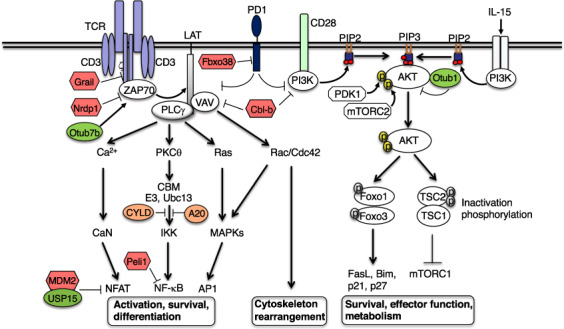
Regulation of T cell signaling by E3 ligases and DUBs. Grail and Cbl-b mediate ubiquitin-dependent degradation of TCR ζ chain and upstream signaling factors, VAV and PI3K, to negatively regulate TCR and CD28 signaling. Non-degradative ubiquitination of ZAP70, a negative mechanism of its regulation, is catalyzed by the E3 Nrdp1 and counteracted by the DUB Otud7b. MDM2 and Peli1 mediates ubiquitin-dependent degradation of NFAT1 and NF-kB c-Rel, respectively, to control these downstream pathways. The DUB USP15 stabilizes MDM2 in activated T cells and functions together with MDM2 in NFAT1 regulation. The CBM complex, composed of CARMA1, BCL10, and MALT1, associates with the K63-specific E2 Ubc13 and an E3 and catalyze K63 ubiquitination required for activation of IKK. This signaling step is negatively regulated by the DUBs CYLD and A20. The DUB Otub1 inhibits K63 ubiquitination and activation of AKT and controls a major metabolic signaling pathway of T cells. E3 ligase Fbxo38 mediates ubiquitin-dependent degradation of the coinhibitory receptor PD1, thereby promoting T cell responses in cancer immunity

Upon engagement by an antigen, TCR initiates receptor-proximal signaling events, including activation of the protein tyrosine kinase Lck and phosphorylation of the immunoreceptor tyrosine-based activation motifs (ITAMs) in CD3 and ζ chains of the TCR complex. Phosphorylation of the ITAMs leads to recruitment and activation of an ITAM-binding protein tyrosine kinase, ZAP70, which in turn amplifies the TCR signal by phosphorylating scaffold proteins, including LAT and SLP76.^68^ These phosphorylated scaffold proteins mediate recruitment and activation of key signaling components that target different downstream signal cascades, leading to activation of transcription factors of the NFAT, AP1 and NF-κB families that cooperatively induce the expression of genes involved in T cell activation, survival, proliferation, and differentiation (Fig. 2).

The TCR-proximal signaling events are tightly controlled by ubiquitination, which mediates degradation or functional inactivation of key components of the TCR signaling pathway, such as CD3ζ, ZAP70, phospholipase C gamma 1 (PLCγ1), PI3 kinase (PI3K), and protein kinase C theta (PKCθ).^5^ A well-characterized E3 ligase involved in the regulation of TCR signaling is Cbl-b, which targets PI3K and the guanine nucleotide exchange factor VAV and is crucial for maintaining T cell tolerance and preventing autoimmunity^69,70^ (Fig. 2). Cbl-b deficiency reduces the activation threshold of T cells and renders T cells hyper-responsive to TCR stimulation even in the absence of CD28 ligation, causing development of autoimmune diseases in mice.^71,72^ Consistently, Cbl-b-deficient CD8 T cells display markedly enhanced activity in rejecting transplanted and spontaneous tumors.^73,74^ Cbl-b deficiency also renders T cells resistant to inhibition by Treg cells and the immune checkpoint PD-L1.^75–77^ Because of the enhanced activation and antitumor responses of Cbl-b-deficient T cells, Cbl-b has become a focus for understanding the mechanisms of antitumor immunity and an attractive cancer immunotherapy target being exploited in preclinical studies and clinical trials.^78,79^ Another E3 ubiquitin ligase Grail (RNF128) has been shown to negatively regulate T cell activation and maintain T cell anergy and tolerance.^80,81^ Grail inhibits TCR-proximal signaling by targeting CD3ζ for ubiquitin-dependent degradation of CD3ζ, and Grail deficiency impairs T cell tolerance and sensitizes mice for autoimmune diseases^82^ (Fig. 2). In CD8 T cells, Grail also mediates ubiquitin-dependent degradation of IL-21 receptor (IL-21R), and Grail-deficient CD8 T cells show enhanced antitumor reactivity and functionality, involving enhanced IL-21R expression and signaling.^83^

Non-degradative ubiquitination of ZAP70 is a mechanism that negatively regulates its activation and TCR signaling^84,85^ (Fig. 2). The E3 ubiquitin ligase NRDP1 conjugates K33-linked polyubiquitin chains to ZAP70, which facilitates recruitment of ubiquitin-binding tyrosine phosphatases, Sts1 (Ubash3b) and Sts2 (Ubash3a),^84^ known to dephosphorylate and inactive ZAP70.^86^ A DUB, Otud7b, deubiquitinates ZAP70 to prevent its association with Sts1 and Sts2, thereby positively regulating TCR signaling.^85^ NRDP1 deficiency selectively promotes CD8 T cell activation and cytokine production.^84^ Since CD8 T cells play a central role in tumor immunity, it will be important to examine whether targeting NRDP1 promotes antitumor immunity and can be exploited as an approach for cancer immunotherapy.

Ubiquitination also regulates signaling steps involved in activation of individual downstream pathways.^5^ In this regard, the NF-κB signaling pathway has been extensively studied for ubiquitin-dependent regulation. Non-degradative ubiquitination, particularly K63 ubiquitination, positively regulates TCR-stimulated activation of IKK and its downstream transcription factor NF-κB. TCR-stimulated activation of PKCθ triggers the assembly of an intermediate signaling complex, composed of the scaffold protein CARMA1 (CARD11), the adapter Bcl10, and the paracaspase MALT1; this so-called CBM complex activates an E3 ubiquitin ligase that functions together with a dimeric E2 enzyme, Ubc13/Uev1A, to catalyze conjugation of K63-linked polyubiquitin chains onto BCL10, MALT1, as well as components of IKK and its activating kinase TAK1.^5,87,88^ The ubiquitin chains conjugated to BCL10 and MALT1 serve as a platform that facilitate the recruitment and activation of TAK1 and IKK.^5^ The ubiquitin-dependent activation of IKK/NF-κB signaling is subject to tight regulation by DUBs, including CYLD and A20^89–91^ (Fig. 2). CYLD deficiency causes spontaneous IKK/NF-κB activation, associated with aberrant T cell activation and development of intestinal inflammation.^89^ T cell-conditional deletion of A20 sensitizes CD8 T cells for activation, and the A20-deficient CD8 T cells display increased production of effector cytokines and heightened antitumor activity.^92,93^ In addition to controlling NF-κB signaling, A20 also functions in a DUB-independent manner to prevent cell death induction by TNF.^57^

NF-κB signaling is also subject to negative regulation by K48 ubiquitination. In response to TCR/CD28 costimulation, the E3 ubiquitin ligase Peli1 catalyzes K48 ubiquitination and proteasomal degradation of an NF-κB member, c-Rel, thereby controlling T cell activation and preventing autoimmunity^94^ (Fig. 2). The Peli1-deficient CD4 and CD8 T cells are hyper-responsive to TCR/CD28 stimulation for production of IL-2 and IFNγ and display reduced sensitivity to inhibition by Treg cells and TGFβ.^94^ The Peli1-deficient mice display stronger tumor-suppressive ability compared to wildtype control mice, associated with enhanced tumor infiltration with CD8 effector T cells (unpublished data). Another E3 ligase, MDM2, negatively regulates TCR-stimulated NFAT activation through targeting NFATc2 (NFAT1) for ubiquitin-dependent degradation.^95^ MDM2 deletion or pharmacological inhibition promotes NFATc2 activation and IFNγ production in T cells. Interestingly, MDM2 itself is also mediated by ubiquitin-dependent degradation in activated T cells, and this process is counter-regulated by an MDM2-binding DUB, USP15. By deubiquitinating and stabilizing MDM2, USP15 facilitates the function of MDM2 in NFATc2 regulation^95^ (Fig. 2). Therefore, USP15 deficiency promotes NFATc2 activation, causes increased T cell responses and function in antitumor immunity.^95^ Several other E3 ubiquitin ligases and DUBs have been shown to regulate TCR signaling.^5,96^

## Costimulatory and coinhibitory signaling

While the TCR provides a primary and antigen-specific signal for T cell activation, costimulatory signals are also required for initial activation and recall responses of T cells. CD28 is a major costimulatory receptor for naïve T cell activation, which potentiates the TCR signaling and lowers the threshold of T cell activation.^65^ CD28 has two ligands, CD80 (B7-1) and CD86 (B7-2), which are expressed on activated APCs particularly DCs. Both CD28 and B7 form a large family of structurally related proteins with costimulatory or coinhibitory functions.^97^ CTLA4 and PD1 are members of the CD28 family that serve as key coinhibitory receptors and are also known as immune checkpoints.^2^ Major breakthrough in cancer immunotherapy came from the successful treatment of advanced cancer patients with the ICIs anti-CTLA4 and anti-PD-1.^67^ Recent studies have also provided new insight into the mechanisms that regulate the fate and function of these immunosuppressive receptors. In particular, an E3 ubiquitin ligase, Fbxo38, has been reported to interact with PD1 and mediate its K48 polyubiquitination and subsequent proteasomal degradation.^98^ T cell-conditional knockout of Fbxo38 results in increased surface expression of PD1 in T cells and impairs antitumor immunity, leading to more rapid tumor growth^98^ (Fig. 2). Another E3 ligase, c-Cbl, has been shown to interact with the cytoplasmic tail of PD1 and mediate ubiquitin-dependent PD1 degradation in the tumor microenvironment.^99^ Ubiquitination and deubiquitination also regulate the stability of PD1 ligand 1 (PD-L1) in tumor cells, which contributes to the regulation of T cell function and antitumor immunity.^100,101^

Another family of T cell costimulatory/coinhibitory receptors includes members of the TNFR superfamily, many of which are induced along with T cell activation and play a particularly important role in regulating effector and memory T cell responses.^102–104^ Among the costimulatory receptors of the TNFR superfamily are OX40 (TNFRSF4), 4-1BB (CD137, TNFRSF9), GITR (CD357, TNFRSF18), CD27 (TNFRSF7), and TNFR2 (TNFRSF). Engagement of these receptors by their ligands from the TNF superfamily enhances effector T cell generation and functionality and facilitates memory development and recall responses. Most, if not all, of the TNFR family of costimulatory receptors also trigger the activation of noncanonical NF-κB pathway, which is integral for the effector and memory T cell responses.^105^ Agonistic monoclonal antibodies for these receptors, particularly OX40 and 4-1BB, promote antitumor T cell responses either alone or in combination with other cancer immunotherapy strategies, such as immune checkpoint blockade, cytokine therapy, and radiation therapy.^106,107^ In addition to extensive preclinical studies, several early phase cancer immunotherapy clinical trials are being conducted to test the efficacy of anti-OX40 and anti-4-1BB in combination therapies.^106,107^

Signal transduction of the TNFR costimulatory receptors involves recruitment of TNFR-associated factors (TRAFs), which in turn target activation of NF-κB and MAPK pathways.^103^ A hallmark of TRAF-mediated signaling is the critical involvement of protein ubiquitination, particularly the non-degradative K63 and linear ubiquitination.^108^ Two functionally redundant E3 ubiquitin ligases, cIAP1 and cIAP2, catalyze K63 ubiquitination in the TNFR signaling complex, which cooperates with the linear ubiquitin ligase LUBAC to mediate canonical NF-κB activation and prevent cell death induction.^57^ The role of K63 ubiquitination in mediating T cell costimulation by TNFRs has been well demonstrated in the 4-1BB pathway. T cell co-stimulation with a 4-1BB agonist antibody triggers K63 ubiquitination of 4-1BB-associated TRAF2, which is crucial for NF-κB activation and induction of antitumor immunity.^109^ Conversely, the 4-1BB signaling is negatively regulated by two DUBs, CYLD and A20, which physically associate with the 4-1BB/TRAF2 complex.^110^ Overexpression of either CYLD or A20 inhibits NF-κB activation by the 4-1BB agonist antibody, whereas silencing these DUBs enhances NF-κB activation mediated by 4-1BB costimulation in human CD8 T cells.^110^ The role of K63 ubiquitination in 4-1BB-mediated CD8 T cell costimulation is further supported by the finding that CD8 effector and memory T cells expressing a dominant-negative mutant of the E3 ligase cIAP2 (cIAP2^H570A^) have attenuated 4-1BB signaling and impaired survival, demonstrated using a viral infection model.^111^

The expression or signaling function of TNFRs is also negatively regulated by degradative ubiquitination. The E3 ligase NEDD4 mediates ubiquitination and degradation of GITR and negatively regulates T cell-mediated antitumor immunity.^112^ Roquin 1 (Rc3h1) and its paralog Roquin 2 (Rc3h2), E3 ubiquitin ligases and RNA binding proteins, repress the expression of OX40, as well as the CD28 family costimulatory receptor ICOS, by promoting degradation of their mRNAs.^113–115^ Roquins play a crucial role in regulating differentiation of Tfh cells and pathogenesis of autoimmune and inflammatory diseases, but it is unclear whether they also play a role in regulating antitumor immunity.^116^ Ubiquitination also negatively regulates the noncanonical NF-κB pathway. In contrast to its K63-specific E3 function in facilitating TNFR-mediated canonical NF-κB activation, cIAP (cIAP1 or cIAP2) negatively regulates noncanonical NF-κB pathway by catalyzing K48 ubiquitination and degradation of the noncanonical NF-κB-inducing kinase NIK.^105^ This function of cIAP also requires TRAF2 and TRAF3, which function in the assembly of the cIAP-TRAF2-TRAF3 complex and recruitment of NIK. TNFR-stimulated noncanonical NF-κB activation involves ubiquitin-dependent degradation of TRAF3 or TRAF2.^105^ Importantly, pharmacological inhibition of cIAPs or genetic ablation of TRAF2 or TRAF3 causes constitutive activation of noncanonical NF-κB.^117^ Consistently, small molecule inhibitors of cIAPs (or smac mimetics) promote T cell activation and effector function and augment antitumor immunity.^118–120^ These studies implicate cIAP inhibitors as new therapeutic agents in combinatorial cancer immunotherapy.

Noncanonical NF-κB activation by T cell costimulation via TNFRs also plays a role in CD4 T cell differentiation, as demonstrated for the induction of Th9 cells by OX40 costimulation.^121^ Th9 cells form a subset of CD4 effector cells characterized by production of the cytokine IL-9. Although Th9 cells have been extensively studied for their involvement in autoimmunity and inflammation, recent evidence suggests that they also serve as a major subset of CD4 effector T cells mediating antitumor immunity.^122–125^ OX40 ligation potently induces the expression of TRAF6, which is required for noncanonical NF-κB activation and Th9 cell generation.^121^ Since TRAF6 is a K63-specific E3 ligase, it is possible that TRAF6 may activate cIAP via K63 ubiquitination, which allows cIAP to mediate K48 polyubiquitination and degradation of TRAF2 or TRAF3 leading to NIK stabilization and noncanonical NF-κB activation. Ubiquitination also regulates the differentiation of antitumor Th9 cells via autophagy, a catabolic mechanism that removes unnecessary and dysfunctional cellular components.^126^ PU.1, an important transcription factor involved in Th9 cell differentiation, undergoes K63 ubiquitination and degradation through selective autophagy. Selective autophagy employs specific cargo adapters to target proteins, protein complexes, or organelles for degradation.^127^ One major cargo adapter of selective autophagy is p62 (also called SQSTM1), which targets ubiquitinated cargos for autophagic degradation.^127^ Silencing of p62 or genetic deletion of major autophagy components in CD4 T cells enhances PU.1 expression and promotes Th9 differentiation and antitumor immunity in different mouse cancer models.^126^

## Cytokine signaling

In addition to the TCR and costimulatory signals, cytokines play an important role in regulating the nature, magnitude, and persistence of T cell responses. In particular, the common gamma chain (γ_c_ or CD132) family of cytokines, especially IL-2, IL-7, IL-15, and IL-21, have been actively explored for improving the efficacy of cancer immunotherapies.^128^ The receptors of these cytokines share the γc subunit that forms dimeric or trimeric receptor complexes mediating signaling that promotes the survival, proliferation, effector/memory generation and maintenance of T cells.^128,129^ Among the γc family cytokines, IL-15 is unique in that it requires a transpresentation mechanism to engage its receptor for signal stimulation.^130^ IL-15 receptor (IL-15R) is composed of three submits: IL-15Rα, IL-15Rβ (also called IL-2Rβ or CD122), and γc. When produced in supporting cells, such as monocytes and dendritic cells, IL-15 forms a complex with IL-15Rα on the cell surface and is presented to the IL-15Rβγ heterodimer on target cells, particularly CD8 T cells and NK cells, to trigger signaling.^130^ Major signaling events stimulated by IL-15 include activation of the transcription factors STAT5 and the PI3K/AKT survival pathway.^131^ Exogenously administered IL-15 sensitizes the TCR for antigen stimulation, promotes CD8 T cell proliferation and effector function, and also rescues tolerant CD8 T cells in cancer immunotherapy.^132–134^ Recombinant IL-15, particularly IL-15 superagonists (IL-15/IL-15Rα complexes), have shown promise in preclinical experiments and clinical trials of cancer immunotherapies when used in combination with other agents, such as ICIs.^135^

**Fig. 3:**
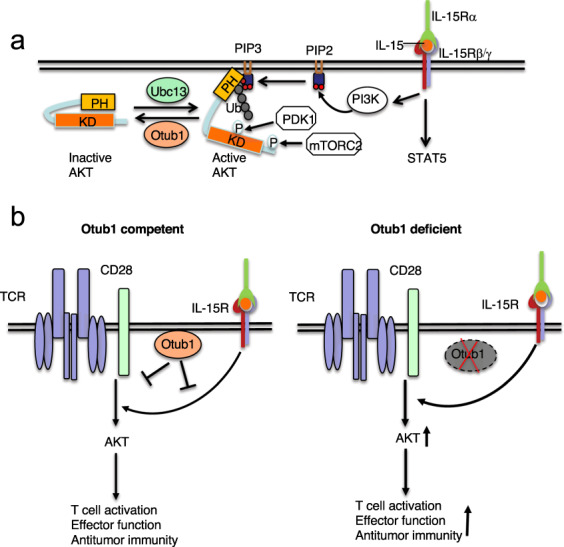
Otub1 serves as a checkpoint of IL-15-mediated CD8 T cell priming by deubiquitination of AKT. **a**, IL-15 stimulates PI3K activation and AKT K63 ubiquitination, both being required for AKT activation. AKT ubiquitination facilitates its binding to PIP3, probably through a conformational change leading to exposure of the PH domain of AKT, which recruits AKT to the membrane compartment, where AKT is phosphorylated and activated by the kinases PDK1 and mTORC2. Otub1 negatively regulates AKT activation through inhibiting its ubiquitination. **b**, IL-15 signal primes CD8 T cells for antigen stimulation, which is controlled by Otub1. In response to IL-15 stimulation, Otub1 is recruited to the membrane compartment, where it inhibits AKT activation induced by both IL-15 and TCR/CD28 signals. Otub1 thus functions as a checkpoint molecule regulating IL-15-mediated CD8 T cell priming. Deficiency in Otub1 causes increased AKT signaling and enhanced CD8 T cell responses in cancer immunity

The role of ubiquitination in regulating signal transduction stimulated by the γc cytokines is poorly studied. Nevertheless, a recent study demonstrated a ubiquitin-dependent mechanism that mediates IL-15-stimulated activation of AKT signaling and CD8 T cell responses in antitumor immunity.^136^ AKT activation is known to be mediated by PI3K, which catalyzes the conversion of the membrane phosphatidylinositol 4,5-bisphosphate (PIP2) to phosphatidylinositol 3,4,5-trisphosphate (PIP3), the latter of which recruits AKT to the membrane compartment for activation by upstream kinases, PDK1 and mTORC2. The PI3K/AKT pathway integrates the TCR/CD28 and IL-15R signals and is negatively regulated by the DUB Otub1 (Fig. 2). IL-15 not only activates PI3K but also induces K63 ubiquitination of AKT, and the ubiquitination enables AKT to bind PIP3 for membrane translocation and activation^136^ (Fig. 3a). Notably, AKT ubiquitination occurs in its pleckstrin homology (PH) domain, which is known to mediate PIP3 binding. Since inactive AKT exists in a closed conformation involving intramolecular interaction between its kinase domain and PH domain,^137^ it is likely that ubiquitination causes AKT conformational changes leading to exposure of the PH domain for PIP3 binding (Fig. 3a). IL-15-stimulated AKT activation is negatively regulated by Otub1, which physically interacts with AKT and prevents its K63 ubiquitination and membrane translocation.^136^ Interestingly, in IL-15-exposed cells, Otub1 is relocated to the membrane compartment, a mechanism that enables Otub1 to inhibit AKT ubiquitination and activation induced by both IL-15 and TCR signals (Fig. 3b). Otub1 deficiency greatly enhances antigen-specific CD8 T cell responses and promotes their metabolic reprogramming and effector function in antitumor immunity.^136^ These studies suggest that IL-15 primes CD8 T cells for antigen-specific responses, which is controlled by a checkpoint, Otub1 (Fig. 3b). The potential for targeting Otub1 in cancer immunotherapy is demonstrated by the finding that inducible deletion of Otub1 in tumor-bearing mice profoundly enhances tumor rejection through unleashing the activity of CD8 T cells as well as NK cells.^136^ Furthermore, analysis of human skin cutaneous melanoma database reveals a remarkable inverse correlation between Otub1 expression levels and the abundance of CD8 effector T cell gene signature and patient survival.^136^

## Ubiquitination regulation of Treg function and immune tolerance

Treg cells are a subset of CD4 T cells that express the lineage transcription factor Foxp3 and have potent immunosuppressive functions.^138^ The majority of Treg cells are developed in the thymus (thymus-derived Treg or tTreg), but Treg cells can also be generated in the periphery (pTreg) or induced in vitro from naïve CD4 T cells (iTreg) cells.^138^ While Treg cells maintain peripheral immune tolerance to prevent the development of autoimmunity, they also serve as a major cell population that suppresses antitumor immunity in the tumor microenvironment.^139^ Treg depletion has been shown to promote antitumor immune responses in preclinical studies, and additional Treg-targeting methods have been actively explored for improving the efficacy of cancer immunotherapy.^139^ Rapid progresses have also been made in understanding of the molecular basis of Treg regulation, providing insight for further improving therapeutic approaches. Ubiquitination has been established as a crucial mechanism that regulates the development and immunosuppressive function of Treg cells.^5,96^

## Nondegradative ubiquitination

A role for nondegradative ubiquitination in regulating Treg development was initially suggested by the finding that mice deficient in CYLD, a DUB cleaving K63 and linear ubiquitin chains, have profoundly increased frequencies of thymic and peripheral Treg cells.^140,141^ Similar results were obtained with mice expressing a nonfunctional CYLD splice variant, CYLD(ex7/8).^142^ This function of CYLD involves, in part, inhibition of ubiquitin-dependent NF-κB signaling,^140^ which is known to promote tTreg development.^143^ In addition, CYLD regulates signaling from IL-2R and TGFβR known to be crucial for Treg generation.^140,141^ A20, another DUB that controls NF-κB signaling via inhibition of K63 ubiquitination, also regulates thymic development of tTreg cells.^144^ Nondegradative ubiquitination also regulates the function of transcription factors involved in induction of Foxp3 expression. One example is ubiquitination of TGFβ-inducible early gene 1 (TIEG1), a Foxp3 transactivator involved in iTreg cell development.^145^ Itch-mediated monoubiquitination of TIEG1 appears to promote its transcriptional activity in Foxp3 gene induction.

K63 ubiquitination plays a critical role in mediating the stability and immunosuppressive function of established tTreg cells. Treg-specific deletion of a K63-specific E2 ubiquitin-conjugating enzyme, Ubc13, impairs in vivo immunosuppressive function of Treg cells and causes aberrant T cell activation and autoimmune symptoms.^146^ The Ubc13-deficient Treg cells are sensitized for acquiring Th1- and Th17-like inflammatory T cell phenotypes under lymphopenic and inflammatory conditions. The function of Ubc13 in Treg cells is mediated through ubiquitin-dependent activation of IKK and involves induction of SOCS1, a pivotal suppressor of proinflammatory cytokine receptor signaling.^146^ TRAF3, a potential K63 ubiquitin ligase, is also involved in the regulation of Treg function. Treg-specific TRAF3 deletion in mice partially impairs the immunosuppressive function of Treg cells, causing an increase in the frequency of Th1-like effector T cells under homeostatic conditions. TRAF3 is also important for antigen-stimulated generation of follicular Treg cells, a subset of Treg cells that functions in germinal centers to suppress Tfh and B cells and, thereby, inhibit antibody responses.^147^ TRAF3 is required for ERK MAPK activation, although it is unclear whether this function involves its K63 E3 ligase activity. Another TRAF family member, TRAF6, also plays an important role in regulating Treg function.^148^ TRAF6 conjugates K63 ubiquitin chains to Foxp3, which mediates proper localization of Foxp3, thereby facilitating its transcriptional function in Treg cells.^148^ Importantly, Treg-conditional TRAF6 knockout mice display increased antitumor immunity, although it remains to be examined whether deletion of Ubc13 or TRAF3 in Treg cells also promotes antitumor immunity.

In addition to K63 ubiquitination, linear ubiquitination regulates signal transduction by nondegradative mechanisms.^149^ Linear ubiquitination is specifically catalyzed by linear ubiquitin chain assembly complex (LUBAC), a multi-subunit E3 composed of HOIL-1L (also called RBCK1), HOIP (also called RNF31), and SHARPIN. A role for linear ubiquitination in regulating Treg function is suggested by a study employing Treg-conditional RNF31 knockout mice.^150^ Treg-specific deletion of RNF31 causes massive loss of Treg cells and development of severe autoimmune and inflammatory pathology. Despite the demonstrated function of LUBAC in preventing TNF-induced apoptosis and necroptosis,^149^ the massive loss of Treg cells in Treg-conditional RNF31 knockout mice cannot be rescued by antibody-mediated TNF neutralization, suggesting a yet-to-be defined mechanism in RNF31-mediated Treg maintenance.^150^ A more recent study shows that RNF31 is also required for maintaining human Treg cell stability and function.^151^ Treg cells with RNF31 knockdown acquires a Th1-like phenotype, characterized by IFNγ production. RNF31 interacts with and stabilizes Foxp3 and catalyzes conjugation of multi-monoubiquitination chains onto Foxp3, suggesting an atypical function of RNF31.^151^ It remains to be investigated how monoubiquitination stabilizes Foxp3 and how LUBAC-mediated linear ubiquitination regulates Treg cell survival and function.

## Degradative ubiquitination

The development and function of Treg cells are also subject to regulation by degradative types of ubiquitination. Ubiquitination is a major mechanism that regulates the stability of Foxp3, a master transcription factor of Treg cells.^152,153^ Ubiquitin-dependent degradation of Foxp3 is strongly induced when Treg cells are exposed to proinflammatory cytokines, the TLR4 ligand LPS, or heat shock stress conditions, which induce the recruitment of Foxp3 to the E3 ubiquitin ligase Stub1 for K48 ubiquitination.^154^ Stub1 knockdown stabilizes Foxp3 and increases the suppressive function of Treg cells, whereas Stub1 overexpression impairs the suppressive function of Treg cells both in vitro and in vivo.^154^ Stub1, together with Cbl-b, also mediate ubiquitin-dependent degradation of Foxp3 in thymic Treg precursor cells, thereby negatively regulating the development of tTreg cells.^155^ Cbl-b deficiency partially rescues the tTreg development defect in CD28-knockout mice. It is thought that in response to TCR/CD28 stimulation, Stub1 initiates Foxp3 ubiquitination, and the ubiquitin chains of Foxp3 facilitate the recruitment of Cbl-b via its ubiquitin-association (UBA) domain, leading to enhanced Foxp3 ubiquitination.^155^ In contrast to its negative role in tTreg regulation, Cbl-b appears to positively regulate the development of iTreg cells.^156^ Cbl-b deficiency impairs the generation of iTreg cells from CD4 T cells due to attenuated Foxp3 induction by the TCR/CD28 and TGFβ signals.^156^ Cbl-b promotes Foxp3 gene induction by facilitating activation of the Foxp3-inducing transcription factors Foxo3a and Foxo1; this function of Cbl-b involves inhibition of TCR/CD28-stimulated activation of AKT, a pivotal negative regulator of Foxo3a and Foxo1.^156^ Another E3 ligase, GRAIL, is upregulated in Treg cells and involved in the regulation of Treg function.^82,157^ GRAIL deficiency does not affect the generation of tTreg or iTreg cells, but impairs their suppressive function.^82^

The tumor suppressor von Hippel-Lindau (VHL) serves as the substrate-recognition subunit of an E3 ubiquitin ligase complex that mediates ubiquitin-dependent degradation of hypoxia-inducible factors (HIFs), including HIF1α and HIF2α.^158^ In addition to hypoxia, many other factors can induce the expression of HIFs in immune cells, such as cytokines and TCR signals.^159^ HIFs play an important role in mediating the development, metabolic activity, and function of conventional T cells.^160^ In particular, HIF1a has been shown to drive CD8 T cell migration and effector function in antitumor immunity.^161,162^ VHL deficiency causes HIF1a accumulation in CD8 T cells and enhances their effector functions in anti-viral and antitumor immunity.^161^ Interestingly, HIF1a accumulation in VHL-deficient Treg cells impairs their suppressive function and causes conversion of Treg cells into Th1-like inflammatory T cells, which are associated with development of autoimmune and inflammatory symptoms in mice.^163^ HIF1a has also been shown to attenuate Treg development by targeting Foxp3 to the VHL E3 ligase complex for ubiquitin-dependent degradation.^152^ These findings suggest that targeting VHL may promote antitumor immunity by both stimulating the effector T cell function and attenuating Treg function.

In line with the function of degradative ubiquitination in Treg regulation, DUBs opposing degradative ubiquitination have been shown to regulate Treg development or function. A TGFβ-induced DUB, USP44, deconjugates K48 ubiquitin chains from Foxp3 and stabilizes Foxp3, which is important for maintaining Treg function.^164^ USP44 physically associates with and functionally cooperates with USP7,^164^ another DUB known to deubiquitinate and stabilize Foxp3.^165^ Mice with Treg-specific USP44 deficiency display enhanced antitumor immunity, implicating USP44 as a potential target for cancer immunotherapy.^164^ Another DUB required for maintenance of Treg stability and suppressive function is USP21.^166^ Treg-specific deletion of USP21 in mice reduces the level of Foxp3 and perturbs the expression of Treg signature genes, causing aberrant T cell activation and autoimmune symptoms. The USP21-deficient Treg cells acquires a Th1-like effector T cell phenotype capable of producing the proinflammatory cytokine IFNγ. It is unclear whether USP21 serves as a DUB of Foxp3 or regulates the level of Foxp3 via an indirect mechanism. In human Treg cells, USP21 stabilizes GATA3,^167^ a transcription factor that mediates Foxp3 expression and is required for the stability and suppressive function of Treg cells.^168^ However, USP21 deletion in murine Treg cells has no obvious effect on GATA3 protein stability or expression level, indicating functional redundancy in the murine system.^166^

## Ubiquitin regulation of NK cell function

Like CD8 T cells, NK cells serve as cytotoxic effector cells mediating destruction of pathogen-infected cells and tumor cells.^169^ However, NK cells are innate lymphocytes that recognize target cells via mechanisms independent of TCR and not restricted by MHC, and they complement the function of CD8 cytotoxic T cells. NK cells express a range of stimulatory and inhibitory receptors, and whether NK cells kill target cells depends on the relative expression level of ligands for stimulatory and inhibitory receptors on target cells. NK cells also act as regulatory cells that modulate dendritic cell function and promote T cell responses.^170^ In tumor microenvironments, NK cells produce the chemokines CCL5 and XCL1, which facilitates recruitment of cDC1 to tumor sites, where cDC1 cells take up tumor antigens and cross present the antigens to tumor-specific CD8 T cells to promote antitumor T cell responses.^171^

**Fig. 4:**
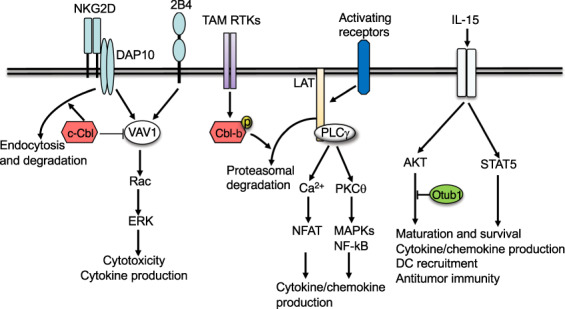
Regulation of NK cell activation by ubiquitination. Cbl-b is an E3 that mediates the inhibitory function of TAM family of receptor tyrosine kinases (RTKs). Upon activation by their ligands, TAM RTKs phosphorylate and activate Cbl-b, which mediates ubiquitin-dependent degradation of a key signaling adapter, LAT, thereby inhibiting activation of several signaling pathways involved in the induction of cytokines and chemokines. Another E3, c-Cbl, negatively regulates NK cell activation by two stimulatory receptors, NKG2D and 2B4, which involves inhibition of VAV1 via non-degradative ubiquitination and ubiquitin-dependent NKG2D endocytosis and degradation. The DUB Otub1 negatively regulates NK cell maturation, chemokine production, and antitumor functions by negatively regulating IL-15-stimulated AKT signaling

Ubiquitination plays an important role in NK cell regulation (Fig. 4). The E3 ubiquitin ligase Cbl-b is a potent negative regulator of NK cell function in cancer immunity. Genetic ablation of Cbl-b or targeted inactivation of its E3 ligase activity does not affect NK cell development but renders NK cells hyper-responsive to activation and capable of spontaneously rejecting metastatic tumors.^172^ Mechanistically, Cbl-b functions in the pathway of the TAM family of receptor tyrosine kinases, including Tyro3, Axl, and Mer, known to inhibit NK cell function. Upon ligand stimulation, TAM receptors become ubiquitinated by Cbl-b, which appears to regulate TAM receptor internalization, but how ubiquitination regulates TAM function is unclear.^172^ A more recent study suggests that Cbl-b serves as a downstream factor mediating NK cell inhibition.^173^ Upon activation by their ligand, TAM receptors mediate tyrosine phosphorylation and activation of Cbl-b, which in turn inhibits NK cell activation by mediating degradation of LAT1, a key signaling adapter downstream of NK cell activating receptors^173^ (Fig. 4). These findings suggest that targeting the TAM/Cbl-b inhibitory pathway provides a new approach to unleash the function of NK cells and may promote antitumor immunity.

Like Cbl-b, c-Cbl plays a role in regulating NK cell activation. In particular, c-Cbl negatively regulates NK cell activation stimulated by co-ligation of two stimulatory receptors, NKG2D and 2B4^174^ (Fig. 4). This function of c-Cbl involves ubiquitination of Vav1, a key signaling factor mediating NK cell co-stimulation by NKG2D and 2B4. It appears that c-Cbl mediates nondegradative ubiquitination of phosphorylated (activated) VAV1 and inhibits VAV1 signaling function.^174^ Another mechanism of c-Cbl action is to promote the endocytosis and degradation of NKG2D stimulated by its ligand, MHC class I-related chain A (MICA).^175^ Interestingly, this function of c-Cbl is not seen when NK cells are stimulated by another ligand of NKG2D, UL16-binding protein 2 (ULBP2). Consistently, MICA, but not ULBP2, stimulates c-Cbl phosphorylation, suggesting ligand-specific function of c-Cbl. Since NKG2D functions as a complex with its adapter DAP10, it is unclear whether c-Cbl ubiquitinates NKG2D or DAP10.^175^ Nevertheless, another study suggests that ubiquitination of DAP10 contributes to the endocytosis and degradation of NKG2D in human NK cells.^176^ This study also suggests that although DAP10 ubiquitination facilitates NKG2D endocytosis and lysosomal degradation, the receptor endocytosis is also required for activation of ERK signaling and NK cell effector functions, suggesting paradoxical roles.^176^

Among other E3s known to regulate NK cell function is NK lytic-associated molecule (NKLAM), a ring finger protein involved in the cytolytic function of NK cells.^177,178^ NKLAM deficiency in mice does not affect NK cell development or maturation, but impairs the cytotoxic activity and cytokine production of NK cells, reducing the ability of mice to control melanoma metastases.^179^ Precisely how NKLAM regulates NK cell cytotoxicity is unclear. Although it has been shown to mediate ubiquitination of uridine kinase like-1, the functional significance remains unclear.^178^ A TRIM family E3, TRIM29, has been shown to be induced along with NK cell activation by the cytokines IL-12 and IL-18 and negatively regulate NK cell activation.^180^ NK cell-specific deletion of TRIM29 enhances IFNγ production by NK cells and promotes antiviral immunity.^180^ TRIM29 mediates ubiquitin-dependent degradation of TAB2, a regulatory subunit of the protein kinase TAK1 that is required for IFNγ induction by IL-12 and IL-18.

Several DUBs have been shown to regulate NK cells. As discussed above, Otub1 is a DUB that regulates CD8 T cell homeostasis and antitumor responses through controlling IL-15R signaling and IL-15-mediated CD8 T cell priming.^136^ Consistent with the role of IL-15 in NK cell regulation, Otub1 is also important for regulating the maturation and activation of NK cells (Fig. 4). Inducible deletion of Otub1 in adult mice using the CreER system markedly increases the frequency of mature NK cells.^136^ Upon in vitro activation by cytokines, Otub1-deficient NK cells display increased production of the cytotoxic effector molecule granzyme B and the chemokine CCL5, the latter of which is known to mediate cDC1 recruitment. Consistently, the inducible Otub1 ablation greatly enhances antitumor immunity, characterized by increased numbers of tumor-infiltrating T cells, NK cells, and cDC1, and the intratumoral cDC1 increase in Otub1-deficient mice is dependent on NK cells.^136^ Otub1 regulates NK cell function by inhibiting IL-15-stimulated AKT ubiquitination and activation. Another study suggests that AKT promotes granzyme B gene expression by promoting deubiquitination and stabilization of XBP1s, a transcription factor that cooperates with T-bet in mediating granzyme B gene transactivation.^181^ These findings implicate Otub1 as an intriguing target to be exploited for cancer immunotherapy.

Another DUB, A20, is crucial for maintaining the survival and homeostasis of NK cells.^182^ NK cell-conditional A20 knockout mice have severe NK cell lymphopenia, although the residual A20-deficient NK cells in the spleen are hyper-proliferative and display an increased level of IFNγ production. The massive loss of NK cells in NK-conditional A20 knockout mice is partially due to increased sensitivity of A20-deficient NK cells to TNF-stimulated cell death. In addition, A20 negatively regulates mTORC1 activation in NK cells, and the elevated mTORC1 activity, together with enhanced TNF sensitivity, appear to be responsible for the NK cell death in the NK-conditional A20 knockout mice.^182^ A20 contains an DUB catalytic domain and two ubiquitin-binding zinc fingers (ZFs), and emerging evidence suggests that ubiquitin binding is a crucial mechanism mediating A20 function.^57^ The NK-regulatory function of A20 is also dependent on its ubiquitin-binding ZFs.^182^ Given the crucial role of NK cells in cancer immunity, targeting ubiquitination pathways represent an attractive approach in cancer immunotherapy.

## Ubiquitin regulation of macrophage function

**Fig. 5:**
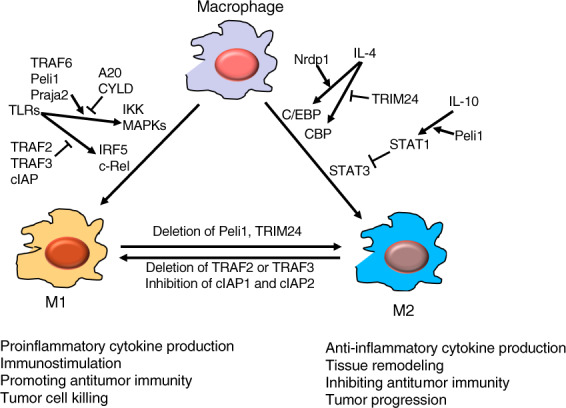
Regulation of macrophage activation and polarization by ubiquitination. E3 ligases TRAF, Peli1, and Praja2 promote TLR-stimulated proinflammatory cytokine expression and M1 polarization of macrophages, which is opposed by the DUBs A20 and CYLD. TRAF2, TRAF3, and cIAP form an E3 complex mediating ubiquitin-dependent degradation of IRF5 and c-Rel, transcription factors mediating induction of proinflammatory cytokine genes and M1 differentiation. E3 ligase Nrdp1 promotes IL-4-induced M2 gene expression by mediating K63 ubiquitination and activation of the transcription factor C/EBPβ. TRIM24 inhibits M2 differentiation through ubiquitinating the acetyltransferase CBP, a mechanism that facilitating CBP-mediated acetylation and attenuation of the M2-promoting transcription factor STAT6. In addition to its M1-promoting function, Peli1 negatively regulates IL-10-induced M2 differentiation. In this function, Peli1 ubiquitinates IRAK1 to trigger its function in activating STAT1, which in turn counteracts the M2-stimulated function of STAT3

Macrophages form a major population of tumor-infiltrating innate immune cells with important roles in regulating tumor microenvironment and antitumor immunity.^183^ Under different conditions, macrophages differentiate into phenotypically different states that are broadly categorized as classically activated (M1) and alternatively activated (M2) macrophages. While M1 macrophages produce various proinflammatory cytokines and chemokines mediating inflammatory responses, M2 macrophages produce anti-inflammatory cytokines important for resolving inflammation and mediating wound healing^183^ (Fig. 5). Tumor-associated macrophages typically resemble M2 macrophages that suppress antitumor immunity via different mechanisms, including inhibition of T cell responses.^183^ These M2-like macrophages also promote tumor progression and resistance to conventional therapies and immunotherapies, thus serving as an important target for cancer therapies. Approaches that target macrophage recruitment, survival, activation and function, have been actively explored for improving cancer immunotherapy.^183,184^ These therapeutic efforts are associated with rapid progress in understanding the molecular mechanisms underlying macrophage polarization and function.

Ubiquitination plays a vital role in regulating macrophage activation through PRRs, which respond to various PAMPs or DAMPs.^5^ Several E3 ubiquitin ligases, including TRAF6 and Peli1, conjugate K63 ubiquitin chains required for signal transduction of TLRs. As indicated in an earlier section, TLR signaling relies on common adapter proteins. The adapter MyD88 transduces signals from all TLRs, except TLR3, whereas another common adapter, TRIF, mediates signaling from TLR3 and TLR4.^5^ TRAF6 mediates signal transduction from MyD88-dependent TLRs by functioning as both an adapter and an E3 ubiquitin ligase.^5^ TRAF6 conjugates K63 ubiquitin chains to target proteins as well as to itself, which is required for recruitment and activation of the ubiquitin-dependent kinase TAK1 and its downstream targets, IKK and MAPKs (Fig. 5). The TRAF6-dependent signaling and proinflammatory gene induction are tightly controlled by DUBs, such as CYLD and A20.^3^ Peli1 functions in both peripheral macrophages and the central nervous system (CNS) resident macrophages, microglia, to mediate TLR-stimulated expression of proinflammatory cytokines and chemokines.^53,185^ Peli1 facilitates NF-κB activation by TRIF-dependent TLRs via mediating K63 ubiquitination of a TRIF-downstream signaling adapter RIP1.^53^ In microglia, Peli1 also mediates MyD88 TLR-induced activation of MAPKs, and this function of Peli1 involves K63 ubiquitination and activation of cIAP, which in turn mediates K48 ubiquitination and degradation of a negative regulator, TRAF3.^185^ Peli1 has also been shown to mediate K63 ubiquitination and activation of the transcription factor IRF5, which contributes to the induction of M1 macrophage polarization.^186^ A more recent study suggests that Peli1 negatively regulates IL-10-induced macrophage polarization to an M2 subtype, M2c, by mediating K63 ubiquitination of IRAK1. IRAK1 ubiquitination promotes activation of the transcription factor STAT1, which suppresses the function of STAT3 in mediating IL-10-induced gene expression and M2c differentiation.^187^ Consistently, myeloid cell-conditional Peli1 knockout mice display increased tumor growth. These studies suggest that Peli1 is an E3 that promotes M1 and inhibits M2 macrophage functions (Fig. 5).

Although TRAF family members are known to function as K63-specific E3s, TRAF2 and TRAF3 have atypical functions in regulating ubiquitin-dependent signaling.^188^ Gene targeting studies reveal that TRAF2 and TRAF3 negatively regulate proinflammatory TLR responses in macrophages.^189^ Myeloid cell-specific deletion of either TRAF2 or TRAF3 sensitizes mice for colitis induction by dextran sodium sulfate, and the TRAF3 deficient mice also spontaneously develop organ inflammation at older ages.^189,190^ Macrophages deficient in either TRAF2 or TRAF3 are hyper-responsive to TLR stimulation in the production of proinflammatory cytokines, including TNFα, IL-1b, IL-6, IL-12, and IL-23.^189^ Mechanistically, TRAF2 and TRAF3 appear to form a K48-specific E3 ubiquitin ligase complex together with cIAP (cIAP1 or cIAP2), which mediate ubiquitin-dependent degradation of transcription factors, including c-Rel and IRF5, involved in proinflammatory cytokine gene induction. Importantly, deletion of TRAF2 in myeloid cells promotes M1-like function of tumor-infiltrating macrophages, which is associated with increased tumor infiltration with IFNγ-producing CD4 and CD8 effector T cells and improved tumor suppression and survival in a mouse model^189^ (Fig. 5). These findings suggest a role for TRAF2, TRAF3, and cIAP in regulating macrophage activation and differentiation and implicate potential therapeutic targets for manipulating tumor-associated macrophage function to favor the induction of antitumor immunity (Fig. 5). The role of Peli1 in facilitating TLR-stimulated TRAF3 degradation is consistent with the proinflammatory function of Peli1.^185^

Several other E3 ligases have been shown to regulate macrophage polarization and function. The E3 ligase Praja2 ubiquitinates malignant fibrous histiocytoma amplified sequence 1 (MFHAS1), a protein involved in regulation of TLR2-stimulated activation of JNK and p38 MAPK pathways.^191^ Praja2 mediates nondegradative ubiquitination of MFHAS1, which facilitates TLR2-stimulated JNK/p38 activation and promotes macrophage polarization to M1 type (Fig. 5). A TRIM family E3, TRIM24, inhibits M2 macrophage differentiation by ubiquitinating the acetyltransferase CREB-binding protein (CBP) and, thereby, facilitating CBP association with and inhibition of, STAT6, a transcription factor mediating induction of M2-associated genes.^192^ CBP-mediated STAT6 acetylation attenuates its transcriptional function in M2 gene induction, which in turn represses M2 polarization. Myeloid cell-specific deletion of TRIM24 promotes tumor infiltration with M2-like macrophages and impairs infiltration with CD4 and CD8 effector T cells, thus increasing the susceptibility of mice for tumor growth^192^ (Fig. 5). Converse to TRIM24, the E3 ligase Nrdp1 promotes M2 macrophage gene induction by IL-4 through mediating K63 ubiquitination and activation of CCAAT/Enhancer-binding protein beta (C/EBPβ).^193^ K63 ubiquitination of macrophage scavenger receptor (MSR1) by an unknown E3 also promotes an anti-inflammatory to proinflammatory shift of IL-4-activated macrophages.^194^ The K63 ubiquitinated MSR1 recruits and activates a signaling complex composed of the ubiquitin-binding kinase TAK1 and its downstream targets MKK7 and JNK, and the JNK signaling axis contributes to pro-inflammatory cytokine induction and phenotypic switch of the IL-4-activated macrophages.^194^ An ATP-binding cassette family member, ABCF1, has recently been shown to function as an E2 conjugating K63 ubiquitination chains to the protein tyrosine kinase Syk and the TRAF member TRAF3, which promotes TLR4 endocytosis and shift from MyD88-dependent to TRIF-dependent signaling, thereby shifting macrophage polarization from M1 to M2.^195^ Collectively, these studies demonstrate crucial roles for ubiquitination in regulating the activation, polarization, and function of macrophages, highlighting the significance of targeting ubiquitin pathways in improving cancer immunotherapy based on modulation of the tumor microenvironment (Fig. 5).

## Ubiquitin regulation of tumor-mediated immunosuppression

Ubiquitination is a well-recognized mechanism that regulates tumor growth and progression as well as tumor microenvironment.^196,197^ In addition, accumulating evidence suggests that ubiquitination also regulates tumor resistance to immune destruction and tumor-mediated immunosuppression. In particular, ubiquitination plays an important role in regulating the fate of PD-L1, a major immune checkpoint molecule that suppresses T cell function via ligation of PD1 on tumor-infiltrating effector T cells.^198^ A cullin-RING E3 family member, cullin3-SPOP, targets PD-L1 for ubiquitin-dependent degradation in tumor cells.^101^ This ubiquitination event is regulated by cyclin D and its catalytic partner cyclin-dependent kinase 4 (CDK4), which mediates phosphorylation and degradation of SPOP, the substrate-binding adapter of the cullin3-SPOP E3 complex.^101^ Pharmacological inhibition of CDK4 or loss-of-function mutations in SPOP attenuate PD-L1 ubiquitination and degradation, resulting in increased PD-L1 expression and decreased tumor-infiltrating lymphocytes in both mouse tumor models and human prostate cancer specimens.^101^ The expression of PD-L1 in cancer cells is also regulated by CKLF-like MARVEL transmembrane domain containing 6 (CMTM6), which stabilizes PD-L1 and suppresses antitumor T cell activity.^199,200^ CMTM6 physically interacts with PD-L1 at the plasma membrane, although precisely how CMTM6 stabilizes PD-L1 is incompletely understood. One study reveals that CMTM6 knockout causes increased ubiquitination of PD-L1, which appears to involve the action of the E3 ligase STUB1,^199^ and another study suggests that CMTM6 prevents lysosomal degradation of PD-L1.^200^

Opposing the action of E3 ligases, specific DUBs mediate PD-L1 stabilization via deubiquitination. A recent study identified COP9 signalosome 5 (CSN5) as a DUB that mediates deubiquitination and stabilization of PD-L1 in cancer cells.^100^ Initially shown to remove the ubiquitin-like modifier Nedd8 from neddylated proteins,^201^ CSN5 was later on found to also possess DUB activity.^202^ Interestingly, CSN5 is required for PD-L1 stabilization and immunosuppression stimulated by the proinflammation cytokine TNFα. TNFα upregulates CSN5 expression through activation of the transcription factor NF-κB p65, which directly transactivates the promoter of the CSN5-encoding gene.^100^ Another DUB, USP22, also stabilizes PD-L1 in tumor cells via deubiquitination and suppresses antitumor immunity in mouse tumor models.^203,204^ While USP22 interacts with PD-L1 and inhibits PD-L1 ubiquitination, this DUB binds and stabilizes CSN5, suggesting that its PD-L1-regulatory function may involve coordination with CSN5.^204^

Ubiquitination also regulates the resistance of cancer cells to immunotherapy. A recent study reveals that a melanoma patient with resistance to PD-1 blockade carries a loss-of-function mutation in the E3 ligase FBXW7.^205^ Consistently, Fbxw7 deletion or mutation in mouse tumors confers their resistance to anti-PD1 therapy. Fbxw7 is required for double-stranded RNA sensing and IFN signaling in tumor cells, which in turn is important for promoting antitumor immunity and immunotherapy. Fbxw7 stabilizes the cytoplasmic RNA sensors, RIG-I and MDA5, although the underlying mechanism is unclear.^205^ Given the role of ubiquitination in regulating different signaling pathways in cancer cells, it is likely that more E3s and DUBs are involved in the regulation of tumor resistance to immune destruction. As such, targeting ubiquitination pathways in tumor cells represents an attractive approach to promote antitumor immunity and cancer immunotherapy.

## Targeting E3s and DUBs for cancer immunotherapy

E3 ubiquitin ligases, which mediate substrate recognition and dictate the specificity of ubiquitination reactions, are important factors for pharmacological targeting in the ubiquitination pathways. Small-molecule inhibitors for a number of E3s have been developed and tested in preclinical models of cancer immunotherapy or combination therapy. Among these are antagonists of the IAP family of E3 ligases, including cIAP1, cIAP2, and X-linked IAP (XIAP), developed as small-molecule mimetics of the endogenous IAP inhibitor Smac (also called Diablo).^206^ Originally developed as drugs to target the apoptosis-inhibitory function of IAPs in cancer cells, the IAP antagonists have now been shown to also potently stimulate antitumor immunity through enhancing innate and adaptive immune responses.^206^ In combination therapies, IAP antagonists significantly increase the efficacy of cancer treatment by other immunological agents or cells, including bacillus Calmette-Guérin (BCG), cytokine-induced killer (CIK) cells, chimeric antigen receptor (CAR) T cells, the NKT cell inducer α-GalCer, TNFα, and PD1 blockade.^207–212^ These preclinical studies highlight the potential for using IAP antagonists as drugs in human cancer immunotherapy, and this intriguing possibility is currently being evaluated in several early phase clinical trials.^206^

Another E3 ligase that is being explored for pharmacological targeting in cancer therapy is MDM2, which promotes tumor growth and progression by mediating ubiquitin-dependent degradation of the tumor suppressor p53 and p53-independent functions.^213^ Recent studies suggest that *MDM2* amplification is correlated with poor clinical outcome and increased rate of accelerated cancer progression, known as hyperprogression, after ICI therapy.^214–216^ Consistently, a small molecule inhibitor of MDM2, AMG-232, sensitizes tumor cells to T cell-mediated killing in vitro.^217^ Another MDM2 inhibitor, APG-115, synergizes with PD1 blockade in a mouse model of cancer immunotherapy through promoting M1 macrophage polarization and T cell activation.^218^ The latter finding is consistent with a prior report that MDM2 negatively regulates antitumor T cell responses.^95^ Another important E3 ligase to be targeted for cancer immunotherapy is the VHL E3 complex, which mediates ubiquitin-dependent degradation of HIF1α and controlling metabolic activities and effector function of T cells.^160^ A small molecule inhibitor has been developed to block the binding of VHL to its substrate HIF1α, although its effect on cancer immunotherapy has not been tested.^219^ Since VHL-mediated HIF1α degradation suppresses the migration and antitumor function of CD8 effector T cells,^161,162^ it is likely that VHL inhibitors will promote CD8 T cell functions in tumor microenvironment.

Small molecule inhibitors for several DUBs have also been developed, and some of them have been shown to inhibit tumor growth in animal models.^220–223^ The most extensively explored DUB for drug development is USP7 with a large number of small molecule inhibitors being developed.^224^ In addition to its well-known function in mediating deubiquitination and stabilization of MDM2,^222^ USP7 stabilizes Foxp3 and a histone acetyl transferase, Tip60, thereby maintaining the function of Treg cells in the immune system.^165,225^ Treatment of mice with different USP7 inhibitors impairs the immunosuppressive functions of Treg cells and promote antitumor immunity.^225–227^ Recently, an anticancer chemotherapy drug, mitoxantrone, has been shown to inhibit USP15, but the potency of USP15 inhibition by this compound is low, and in vivo studies have not been performed.^228^ Given the recent advances in small-molecule screening technologies and DUB structural biology, it is anticipated that specific inhibitors for more DUBs as well as E3s will become available in the coming years.

## Concluding remarks

Ubiquitination has become a well-recognized mechanism that regulates signal transduction involved in a broad range of immune system functions, ranging from antigen processing and presentation, T cell activation and tolerance, and NK cell function to innate immune functions mediated by macrophages. A large number of E3 ubiquitin ligases and DUBs have been characterized in different immune cell types, as well as cancer cells, as pivotal regulators of antitumor immunity and tumor-mediated immunosuppression. An increasing number of preclinical studies have demonstrated the effectiveness of targeting these ubiquitin-associated factors for improving the efficacy of cancer immunotherapy. Given the existence of more than 600 E3s and around 100 DUBs in mammalian cells, the ubiquitin-dependent therapeutic approach offers plenty of opportunities in drug development for cancer treatment. Therefore, targeting ubiquitin-dependent signaling represents an attractive new approach that complements the currently available approaches in cancer immunotherapy, such as the ICIs.

For future studies, it is important to characterize novel E3s and DUBs involved in the regulation of antitumor immunity and elucidate the mechanisms of their functions. In addition to animal models, human primary cells provide an important experimental system for assessing the role of the identified factors in regulating human immune cell functions. For further translating the laboratory discoveries into clinical practices, a major task is to develop specific inhibitors targeting more E3 ligases and DUBs, especially those involved in the regulation of antitumor immunity. The advances in small molecule inhibitor development will in turn promote more preclinical studies as well as clinical trials to characterize ubiquitin-based drugs to be employed in the clinic for cancer immunotherapy. In addition to developing small molecule inhibitors, more effort should also be spent on preclinical studies employing the adoptive cell therapy approach, in which specific E3s and DUBs can be knocked out or knocked down for improving the therapeutic efficacy. This latter approach is particularly attractive for adoptive T cell and NK cell therapies based on chimeric antigen receptors.
